# Iridology: Biological plausibility, clinical evidence, and implications for ophthalmic practice: *A position paper of the Associazione Pazienti Malattie Oculari (APMO)*

**DOI:** 10.1007/s00417-026-07342-w

**Published:** 2026-06-25

**Authors:** Lorenzo Iuliano, Daniele Veritti, Paolo Lanzetta

**Affiliations:** 1 Department of Ophthalmology, San Raffaele Scientific Institute, Vita-Salute University, Milan, Italy; 2 Department of Medicine-Ophthalmology, University of Udine, Piazzale Santa Maria della Misericordia, 33100 Udine, Italy; 3 Azienda Sanitaria Universitaria Friuli Centrale, Udine, Italy; 4 Istituto Europeo di Microchirurgia Oculare - IEMO, Udine, Italy

**Keywords:** Iridology, Iris, Alternative medicine, Diagnostic accuracy, Ophthalmology, Evidence-based medicine, Epistemology

## Abstract

Iridology is an alternative diagnostic practice that claims to identify systemic diseases and organ dysfunction through visual inspection of iris features, including pigmentation patterns, crypts, furrows, and discolorations. Despite its continued presence within complementary and alternative medicine, iridology has not been incorporated into mainstream medical practice. This review critically examines iridology from an ophthalmologic perspective, addressing its historical origins and epistemological foundations, proposed mechanisms, biological plausibility, and clinical evidence. A systematic appraisal of the available literature, including the most recent government-commissioned evidence evaluation, demonstrates a consistent lack of diagnostic accuracy, reproducibility, and pathophysiological rationale. The ethical and clinical implications of iridology use are discussed, with particular attention to the risk of delayed diagnosis and patient misinformation. Based on the totality of evidence, iridology cannot be supported as a diagnostic or screening tool in ophthalmology or general medicine.

## Key messages

**What is known:**

Controlled clinical studies published since 1979 have consistently demonstrated that iridology has no diagnostic accuracy beyond chance level, and no biologically plausible mechanism linking iris features to systemic organ pathology has ever been identified.

**What is new:**

Recent studies, including the most rigorous government-commissioned systematic review to date (Australian NHMRC, 2024), confirm that current evidence does not support the use of iridology in ophthalmic or general medical practice.

The *Associazione Pazienti Malattie Oculari* (APMO) endorses this position and calls for clear, evidence-based communication between ophthalmologists and patients regarding iridology claims.

## Introduction

The iris is a highly specialized ocular structure whose primary functions are regulation of light entry and contribution to optical quality. Advances in ophthalmic imaging and pathology have demonstrated that iris morphology is determined predominantly by genetic, developmental, and limited disease-specific factors [1–3]. 

In contrast, iridology proposes that specific regions of the iris correspond topographically to distant organs and that systemic disease can be diagnosed through iris inspection. Although iridology is occasionally encountered by ophthalmologists through patient inquiries or referrals from alternative practitioners, it remains absent from evidence-based diagnostic frameworks [4]. Given the increasing importance of addressing health misinformation within clinical practice, and the persistence of iridology despite consistently negative evidence, a thorough and scientifically rigorous appraisal is relevant and necessary for the ophthalmic community.

## Methods

This mini-review was conducted as a narrative synthesis of the available literature on iridology. A structured search was performed in PubMed, Scopus, Web of Science and Google Scholar databases up to March 2026, using combinations of the keywords (“iridology” OR “iridodiagnosis”) AND (“iris” OR “systemic disease” OR “alternative medicine”). Priority was given to peer-reviewed original research articles, systematic reviews, multicenter studies, and authoritative guidelines. Reference lists of included articles were manually screened to identify additional relevant studies.

The initial search retrieved 48 records. After removal of 2 duplicates, 46 titles and abstracts were screened, of which 34 were excluded as not relevant to iridology. The remaining 12 full-text articles were assessed for eligibility and supplemented by 3 additional studies identified through manual screening of reference lists, yielding 15 studies included in the qualitative synthesis. Of these, 9 controlled diagnostic-accuracy studies (Tables 1 and 2) were eligible for formal methodological appraisal; the remaining 6 (three computer-aided/machine-learning studies [Table 3] and three iris-constitution genetic-association studies) were analyzed and discussed but were not amenable to diagnostic-accuracy appraisal owing to their design.

**Table 1:** Controlled studies evaluating the diagnostic accuracy of iridology, listed in chronological order

Study (year)	Journal	Condition tested	Design / *n*	Main finding
*Simon et al. (1979)*	*JAMA*	Renal disease	Blinded case-control; *n* = 143 (48 cases, 95 controls); 3 iridologists, 3 ophthalmologists	Sensitivity/specificity at chance level; no discriminatory ability
*Cockburn (1981)*	*Aust J Optom*	Multiple systemic conditions	Controlled blinded study	No diagnostic accuracy beyond chance
*Popescu & Waniek (1986)*	*Oftalmologia*	Mitral stenosis	Case-control; *n* = 23; automated photodensitometry	Significant difference (*p* < 0.05); methodologically limited; small sample; incomplete blinding details
*Knipschild (1988)*	*BMJ*	Gallbladder disease	Blinded case-control; 39 cases vs. 39 controls; 5 leading iridologists	Median validity 51%, sensitivity 54%, specificity 52%; all close to chance
*Buchanan et al. (1996)*	*Compl Ther Med*	Ulcerative colitis, asthma, coronary heart disease, psoriasis	Blinded case-control; *n* = 145; manual and computer-aided analysis	Discrimination between cases and controls no better than chance
*Münstedt et al. (2005)*	*J Altern Complement Med*	Breast, ovary, uterus, prostate, colorectal cancer	Prospective blinded case-control; *n* = 110 (68 cases, 42 controls)	Sensitivity 0.04; iridology of no diagnostic value
*Stearn & Swanepoel (2007)*	*Afr J Tradit Complement Altern Med*	Sensorineural hearing loss (adolescents)	Blinded controlled trial; 100 enrolled (50 cases, 50 controls), ages 15–19; 47 photographs discarded for poor quality, 53 analyzed; single iridologist, Jensen chart	Sensitivity 59%, specificity 81%; statistically significant (*p* < 0.05) but well below audiological screening standards; 47% of images unusable
*Herber et al. (2008)*	*Ophthalmologe*	Colorectal cancer	Blinded; 29 cases vs. 29 controls; 2 iridologists	Detection rate 50–53%; no better than chance
*Aishwarya et al. (2025)*	*Adv Integr Med*	Female reproductive system abnormalities	Cross-sectional, unblinded; *n* = 100; ultrasound reference standard	Sensitivity 92%, specificity 56%; unblinded design; false-positive rate > 40%; cannot validate iridology

*n* number of participants

**Table 2:** Methodological quality of the controlled diagnostic-accuracy studies assessed with the QUADAS-2 tool

Study (year)	Patient selection	Index test	Reference standard	Flow and timing
Simon et al. (1979)	High	Low	Low	Unclear
Cockburn (1981)	High	Low	Low	Unclear
Popescu & Waniek (1986)	High	Unclear	Unclear	Unclear
Knipschild (1988)	High	Low	Low	Low
Buchanan et al. (1996)	High	Low	Low	Unclear
Münstedt et al. (2005)	High	Low	Low	Low
Stearn & Swanepoel (2007)	High	Low	Low	High
Herber et al. (2008)	High	Low	Low	Unclear
Aishwarya et al. (2025)	Unclear	High	Unclear	Unclear

*QUADAS-2* Quality Assessment of Diagnostic Accuracy Studies 2

Risk of bias was judged across four domains as low, high, or unclear, based on the characteristics reported in each primary publication. A high risk of bias in patient selection reflects the case–control design with healthy comparators common to these studies, which tends to inflate apparent discriminative ability. The favorable results came from the studies with unclear or absent masking of the index test (Popescu & Waniek; Aishwarya et al.); where the index test was masked, the most rigorous study (Münstedt et al.) found no diagnostic value, and the one masked study reporting a statistically significant association (Stearn & Swanepoel) nonetheless yielded sensitivity and specificity inadequate for clinical screening

**Table 3:** Computer-aided and machine-learning studies applying iridology to the detection of type 2 diabetes

Study (year)	Journal	Sample / method	Reported performance	Key methodological limitations
Bansal et al. (2015)	Int J Diabetes Dev Ctries	80 subjects (40 diabetic, 40 healthy); left eye only; SVM (RBF kernel); 2-D wavelet (db2) features; ROI cropped to iris “pancreas zone” per iridology chart; 4-fold cross-validation	Overall accuracy 87.50%; best-case sensitivity 0.95, specificity 0.90	Small closed dataset; no independent/external validation; ROI pre-defined by the iridology chart (circularity); authors note misclassification of operated and well-controlled cases and possible reporting bias
Samant & Agarwal (2018a)	J Med Eng Technol	200 subjects (100 diabetic, 100 non-diabetic); five classifiers compared; ROI cropped to iris “pancreas zone” per Jensen chart; 10-fold cross-validation	Best accuracy 89.61% (random forest); sensitivity 92.36%, specificity 90.59%	Described by the authors as a pilot study; closed dataset; no independent validation; ROI pre-defined by the iridology chart, so the model assumes the validity of the framework it tests (circularity)
Samant & Agarwal (2018b)	Comput Methods Programs Biomed	338 subjects (180 diabetic, 158 non-diabetic); five classifiers compared; ROI cropped to iris “pancreas zone” per iridology chart	Best accuracy 89.63% (random forest)	Closed dataset; no independent validation; no prospective design; ROI pre-defined by the iridology chart (circularity)

*SVM* Support vector machine

*ROI* Region of interest

Reported performance refers to internal classification accuracy on the study dataset and should not be equated with validated diagnostic accuracy. The two Samant & Agarwal entries are companion publications by the same group reporting closely overlapping analyses. In all three studies the region of interest was explicitly cropped to the iris “pancreas zone” defined by the iridology chart, so the algorithm presupposes the validity of the map it is intended to evaluate. Across all studies the reference standard was clinically established diabetes, no dataset was independently validated, and no study employed a prospective design with a pre-specified hypothesis

The methodological quality of the controlled diagnostic-accuracy studies (Table 1) was appraised using the QUADAS-2 tool across its four domains [5]. The appraisal was based on the characteristics reported in the primary publications and is summarized in Table 2.

### Historical origins and epistemological foundations

Iridology originated in the 19th century, most attributed to Ignaz von Peczely, a Hungarian physician. According to the canonical account, von Peczely as a child noticed a dark mark appearing in an owl’s iris following a leg fracture and later observed the mark changing as the injury healed. After qualifying as a physician, he extended this observation to hospital patients, publishing the first iris chart in 1881 [4, 6]. 

A contemporary but independent contribution came from Nils Liljequist, a Swedish homeopath who observed changes in his own iris coloration following treatment with quinine and iodine for malaria, publishing an atlas of iris illustrations in 1893. From an epistemological standpoint, Liljequist’s observation is the most scientifically interesting of the founding accounts: iodine and quinine are pharmacologically capable of producing genuine pigmentary changes in iris melanocytes [6]. However, his critical error was the unjustified generalization from a specific drug-induced pigmentary effect to a universal theory that the entire iris encodes the functional state of all body organs. In the early 20th century, Pastor Emanuel Felke embedded iridology within a constitutional typology linked to homeopathic doctrine. In the 1950s, Bernard Jensen, an American chiropractor, expanded European charts for the US market, creating a 90-zone map now widely used in North America [6]. 

A critical epistemological observation is that these charts were never derived from anatomical or physiological investigation. They were constructed through uncontrolled post hoc correlation: practitioners observed patients with known diagnoses, noted iris features they considered relevant, and retrospectively assigned those features to iris zones corresponding to the affected organ. As acknowledged even within the iridology literature itself, “nearly every iris researcher has tried to evolve something special for himself, with the result that varying perceptions and interpretations are current” [6]. No blinding, no prospective design, no control population, and no statistical testing were applied at any stage of chart development. This mode of chart construction, by uncontrolled post hoc correlation rather than anatomical investigation, is shared with other historical diagnostic systems not derived from controlled study (Table 4).

**Table 4:** Epistemological origins of iridology iris charts

Founder	Period / Country	Original observation	Scientific status
Ignaz von Peczely	1860s-1881, Hungary	Single anecdote: dark mark in owl’s iris after leg fracture	No controlled methodology; pure confirmation bias
Nils Liljequist	1864–1893, Sweden	Iris color change after quinine/iodine treatment for malaria	Genuine drug-induced pigmentary change; unjustified generalization to whole-body organ mapping
Pastor Emanuel Felke	Early 1900s, Germany	Constitutional typology linked to homeopathic doctrine (blue = lymphatic; brown = biliary)	Embedded in vitalistic framework; no anatomical basis
Bernard Jensen	1950–1980 s, USA	90-zone chart; nutritional deficiency correlations	Post hoc pattern-matching; failed first blinded controlled test

### Proposed mechanisms and biological plausibility

Iridology suggests that pathological changes in distant organs induce visible alterations in the iris stroma via neural, vascular, or reflex pathways. This concept was formulated by Bernard Jensen as “nerve fibers in the iris respond to changes in body tissues by manifesting a reflex physiology corresponding to specific tissue locations” [4]. From a biological standpoint, this hypothesis lacks any supporting anatomical or physiological mechanism.

The iris develops embryologically from neuroectoderm and mesenchyme, sharing no developmental lineage with most abdominal or thoracic organs [1, 2]. It receives innervation exclusively from the ciliary ganglion and superior cervical ganglion, governing pupillary light reflexes and vascular tone, with no afferent pathways capable of encoding organ-specific pathological states into localized stromal modifications [1, 2]. It also lacks any known vascular or lymphatic connection that could transmit organ-specific disease signals to discrete iris zones [1–3]. 

It is important to distinguish iridology claims from genuine ocular signs of systemic disease. Kayser-Fleischer rings in Wilson disease, Lisch nodules in neurofibromatosis type 1, and arcus senilis in hyperlipidemia each reflect discrete, well-understood pathophysiological mechanisms involving specific molecules or cell types reaching the iris through known anatomical pathways [1–3]. These signs are entirely different in kind from iridology’s claim that the iris encodes the functional state of all organs through a hypothetical reflex map. No credible anatomical, molecular, or physiological mechanism has been demonstrated to support the core premise of iridology [4]. 

One additional argument sometimes advanced by proponents – that shared neuroectodermal embryological origin between the iris and the nervous system supports a functional diagnostic link – represents a misapplication of developmental biology. Shared embryological origin does not imply functional information transmission in adult anatomy; the same reasoning would require that any two neuroectodermal-derived structures reflect each other’s pathology, which is anatomically untenable.

### Clinical evidence and diagnostic accuracy

The body of controlled clinical evidence is limited in volume but consistent in direction. A systematic review by Ernst [4] identified four masked case-control studies: three demonstrated diagnostic accuracy no better than chance [7–9]; one study [10] of mitral stenosis using automated photodensitometry showed a marginally significant difference (*p* < 0.05) between patients and controls, though this relied on a small sample and an automated technique distinct from standard iridologist practice. An additional early controlled study by Cockburn, not captured by Ernst’s review, similarly found no diagnostic accuracy beyond chance across multiple systemic conditions [11]. 

The Database of Abstracts of Reviews of Effects (DARE) critical appraisal of the Ernst review noted limitations (single-author process, incomplete reporting) underscoring that even the primary negative systematic review has methodological boundaries requiring acknowledgement. Ernst subsequently concluded in a dedicated ophthalmology commentary that iridology is not useful and is potentially harmful [12]. 

Subsequent controlled trials have consistently replicated negative findings. Münstedt et al. conducted the most rigorous post-2000 study: a prospective blinded case-control trial in which an experienced iridologist examined 110 subjects (68 with histologically confirmed cancers, 42 controls) without knowledge of their sex or medical history [13]. Iridology correctly identified the primary diagnosis in only 3 of 68 cases, yielding a sensitivity of 0.04: effectively indistinguishable from random assignment. Herber et al. evaluated colorectal cancer detection using two iridologists in a blinded design; cancer detection rates of 50–53% were achieved, equivalent to coin tossing [14]. 

A blinded controlled trial in adolescents with sensorineural hearing loss reached statistical significance (*p* < 0.05) but yielded a sensitivity of only 59% and a specificity of 81%, figures the authors themselves acknowledged were not comparable to established audiological screening. Notably, 47% of iris photographs had to be discarded for inadequate quality, further limiting applicability [15]. 

In 2022–2025, the most comprehensive recent review was conducted by the Australian National Health and Medical Research Council (NHMRC), commissioned by the Australian Department of Health and Aged Care as part of the Natural Therapies Review 2024. Searching multiple databases from inception through May 2022, this systematic review (conducted using the GRADE framework) concluded, with low certainty evidence, that iridology is not an effective diagnostic tool, consistent with all prior systematic reviews. Iridology was not recommended for re-inclusion in Australia’s private health insurance rebates [16]. 

One recent study reported a sensitivity of 92% and specificity of 56% for iridology in detecting female reproductive system abnormalities using ultrasound as the reference standard [17]. However, this study was unblinded, introducing significant ascertainment bias, and a specificity of 56% implies a false-positive rate exceeding 40%, rendering the test clinically unusable as a screening tool. This study cannot be interpreted as validating iridology. Table 1 summarizes all identified controlled trials. Formal appraisal using QUADAS-2 (Table 2) showed that almost all studies carried a high risk of bias in patient selection, as a case-control design with healthy comparators tends to inflate apparent discriminative ability. Notably, the two studies reporting a favorable result were precisely those with unclear or absent masking of the index test, whereas the most rigorous study (Münstedt et al.) found no diagnostic value.

Across the controlled studies, the recurring problem is not merely low positive and negative predictive value, likelihood ratios close to unity, and areas under the ROC curve near 0.5, which leave the post-test probability of disease essentially unchanged from the pre-test probability: the formal signature of a test without clinical utility. A diagnostic method that cannot shift the probability of disease, and whose results are poorly reproducible, cannot be recommended even when isolated studies report apparently favorable sensitivity.

A few remarks are warranted regarding reproducibility, which is a minimum requirement for any diagnostic test. Studies assessing inter- and intra-observer agreement among iridologists have consistently demonstrated poor concordance, both in identifying iris features and in assigning diagnoses [4]. The existence of multiple incompatible iris charts (Jensen, European/German, Constitutional) each assigning different organs to the same iris zones, reflects a fundamental lack of standardization. Notably, the founding figures themselves produced divergent maps despite working from ostensibly the same premise, consistent with chart construction through individual interpretation rather than objective anatomical observation [6]. 

### The eye as a legitimate window on systemic disease

Criticizing iridology must not be mistaken for the claim that the eye holds no information about systemic health. On the contrary, the eye is one of the most powerful sites in medicine for detecting systemic disease, precisely because validated ocular biomarkers are grounded in anatomy, vascular biology, and disease-specific mechanisms rather than in symbolic maps. The retina, as vascularized neural tissue, directly reflects systemic microvascular, neurological, inflammatory, and metabolic processes: diabetic and hypertensive retinopathy, retinal vascular caliber changes, and optic nerve and retinal-layer alterations quantifiable by OCT and OCT angiography are established, reproducible markers of systemic disease with well-defined pathophysiological correlates [1–3, 18, 19]. 

Crucially, even the iris genuinely reflects systemic disease, but through demonstrable mechanisms, not organ-zone correspondences. In diabetes, iris neovascularization and microvascular leakage (diabetic iridopathy) can be objectively documented by iris fluorescein and indocyanine-green angiography, and correlate with concurrent retinopathy [20–23]. These findings localize to actual, visualizable vessels through known pathophysiology, in direct contrast to iridology’s claim that discrete iris zones encode the state of distant organs. The iris is also the site of numerous legitimate clinical entities recognized by ophthalmology: iris naevi and melanoma, rubeosis iridis, congenital vascular anomalies, vascular tufts, anterior uveitis, pigment dispersion, pseudoexfoliation, traumatic and congenital defects, heterochromia, and medication-induced pigmentation; each assessable by established imaging [24–26]. 

What distinguishes all of these from iridology is not the structure examined but the rigor of the method. Modern ophthalmic imaging (slit-lamp biomicroscopy, colour fundus photography, OCT, OCT angiography, fundus autofluorescence, fluorescein and indocyanine-green angiography, ultra-widefield imaging, adaptive optics, anterior-segment OCT, ultrasound biomicroscopy, and standardized iris photography) generates objective, anatomically grounded biomarkers [27, 28]. Each is validated through standardized acquisition protocols, defined reference standards, masked grading, reproducibility testing, longitudinal follow-up, disease staging, treatment-response assessment, and external validation across diverse populations. This is the evidentiary framework that iridology has never satisfied, and against which its claims must be judged.

### Comparison with established ophthalmic diagnostics

Modern ophthalmic diagnostic modalities, including slit-lamp biomicroscopy, fundus photography, optical coherence tomography (OCT), and fluorescein angiography are grounded in defined anatomical correlates, quantifiable parameters with known inter-operator reproducibility, and validation across large, diverse patient populations with clearly defined reference standards [1–3]. Each technology is directly linked to specific tissue structures, produces measurements amenable to statistical analysis, and has been evaluated against gold-standard reference tests using pre-specified hypotheses and blinded designs.

Iridology lacks all these elements. It has no standardized methodology across schools and practitioners, no objective outcome measures, no validated population-level diagnostic performance data, and no agreed reference standard for what constitutes a positive test. Unlike validated ocular biomarkers of systemic disease (such as Kayser-Fleischer rings, papilledema, or diabetic retinopathy) iridology’s organ zones cannot be anatomically traced to the structures they purport to represent. Its claims diverge sharply from the evidentiary framework applied to contemporary ophthalmic diagnostics [4, 12]. 

### Computer-aided iridology: An emerging but unvalidated development

A contemporary development meriting specific discussion is computer-aided iridology (CAI), which applies digital image processing and machine learning algorithms to iris photographs to automate pattern recognition. A scoping review by Esteves et al. documented significant technological advances in iridological image acquisition and analysis from 2014 to 2019, while also noting persistent methodological heterogeneity across studies [29]. A subsequent methodical review concluded that CAI remains outside accepted medical practice and is not demonstrably more reliable than conventional iridology [30]. 

A growing number of studies have applied machine-learning classifiers to iris images for the detection of systemic disease, most frequently type 2 diabetes, reporting high internal classification accuracies approaching 90% (Table 3) [31–33]. At first sight these figures appear to lend quantitative support to iridological claims; on closer examination, they share three features that preclude such an interpretation. First, in each study the region of interest was pre-defined as the iris “pancreas zone” specified by the iridology chart, so the algorithm is trained to discriminate within the very zone whose validity is in question: a circular design in which a positive result presupposes rather than tests the underlying map. Second, all reported figures derive from internal cross-validation on small, closed datasets, without independent or external validation and without a prospective design against a pre-specified reference standard; the published models are further limited by susceptibility to overfitting, inconsistent image standardization, absence of masking, undefined diagnostic thresholds, and heterogeneous reference standards, and high accuracy on a training set does not constitute clinical validation. Third, the authors of these studies themselves describe their work as preliminary and acknowledge that further investigation is required before iridology can be considered validated for diabetes diagnosis [32, 33]. 

The contrast with validated ophthalmic artificial intelligence is instructive. Algorithms used for diabetic retinopathy screening, OCT interpretation, and glaucoma detection are trained on retinal lesions with established anatomical and pathophysiological correlates and validated prospectively against recognized reference standards in large, diverse populations. The limiting factor for CAI is therefore not the sophistication of the algorithm but the validity of its anatomical premise: a model that learns from an unvalidated map cannot yield a validated diagnosis. The same circular reasoning underlies a series of studies correlating “iris constitution” with genetic markers of vascular disease, which presuppose the validity of the constitutional classification under examination and derive from largely overlapping cohorts studied by a single research group [34–36]. 

Until CAI studies incorporate rigorous prospective designs with pre-specified hypotheses, independent test sets, and comparison against validated reference standards, they cannot be used to support iridology as a diagnostic modality.

## Ethical and clinical implications

The continued promotion of iridology raises several ethical and clinical concerns. Reliance on iridology as a primary or exclusive diagnostic method may delay evidence-based medical evaluation, with potentially serious consequences for conditions where early detection is critical, such as malignancy or endocrine disease [37, 38]. Conversely, false-positive interpretations may generate unwarranted patient anxiety and lead to unnecessary investigations, creating both psychological harm and avoidable economic costs. The use of iris imagery and modern digital equipment to imply diagnostic capability risks conferring an unearned appearance of scientific legitimacy, which may further mislead patients.

Professional organizations consistently state that iridology has no role in medical diagnosis or disease screening. The Australian Government’s Natural Therapies Review 2024 (one of the most rigorous government-level evidence appraisals of any alternative therapy) concluded that iridology should not be eligible for health insurance rebates [16]. From an ophthalmologic perspective, the appropriation of ocular examination by an unvalidated practice risks undermining patient trust in legitimate ophthalmic assessment [12]. 

### Patient communication and the role of the ophthalmologist

Ophthalmologists increasingly encounter patients exposed to health misinformation through digital media and alternative health networks. Addressing iridology requires respectful but clear communication that acknowledges the patient’s perspective while firmly conveying the absence of supporting evidence [37, 38]. 

Effective communication should distinguish validated ocular signs of systemic disease from unsupported iridology claims, without dismissing the patient’s interest in holistic health; explain the specific epistemological weaknesses of iridology, particularly that its diagnostic maps were never derived from controlled anatomical or physiological research, but constructed through uncontrolled post hoc correlation; reinforce the importance of evidence-based screening and not discourage patients from seeking conventional medical evaluation.

This approach aligns with both the ethical obligation to promote informed decision-making and the practical goal of maintaining the therapeutic relationship with patients who may hold alternative health beliefs. The ophthalmologist’s unique position, as both an expert in ocular anatomy and a clinician whose instruments are appropriated by iridology practice, places a particular professional responsibility to address these claims with scientific clarity and empathy.

### Why iridology persists despite negative evidence

Several factors contribute to the persistence of iridology despite consistently negative clinical evidence. Its visual appeal, using a body part (the iris) that is genuinely unique to each individual and aesthetically striking, lends intuitive plausibility. The promise of a non-invasive, painless, rapid assessment of whole-body health addresses a real and understandable patient desire for accessible diagnostics [37, 38]. 

Confirmation bias operates at multiple levels: both practitioners who attribute clinical improvements to iridology-guided interventions, and patients who find that iris descriptions resonate with their subjective health experience, are susceptible to this cognitive distortion [12]. Limited public understanding of what constitutes scientific validation (including the distinction between anecdotal experience and controlled evidence) further sustains the practice. The increasing sophistication of digital imaging and artificial intelligence has given iridology a contemporary technological veneer, which may be misinterpreted as scientific progress. Clinicians who understand these dynamics are better positioned to engage constructively with patients rather than dismissively.

## Conclusions

Iridology is a diagnostic practice whose foundational maps were constructed through uncontrolled post hoc observation, without anatomical, physiological, or embryological basis. Decades of controlled investigation – including the most recent government-commissioned systematic review applying GRADE methodology [16] – have failed to demonstrate diagnostic accuracy beyond chance, and no credible mechanism links iris features to systemic organ pathology.

A scientifically rigorous appraisal must acknowledge several nuances: the evidence base itself is limited in volume and methodological quality; a single study using automated photodensitometry produced one marginally significant finding; and one recent unblinded study reported high sensitivity at the cost of unacceptably low specificity. These exceptions do not alter the overall conclusion but illustrate that further high-quality prospective blinded trials would strengthen the evidentiary record.

Based on the available evidence, the *Associazione Pazienti Malattie Oculari* endorses the following key messages:

- Iridology should not be used or endorsed as a diagnostic or screening tool in ophthalmology or general medicine.
- The epistemological foundations of iridology (chart construction through uncontrolled post hoc correlation) are incompatible with scientific validation regardless of clinical trial results.
- Computer-aided iridology represents a technological advance that has not yet addressed the underlying validity problem and should not be regarded as validated.
- Patient inquiries should be addressed with empathy, scientific clarity, and a clear distinction between genuine ocular signs of systemic disease and unsupported claims.
- Ophthalmologists have a professional responsibility to safeguard the scientific integrity of ocular diagnostics and to protect patients from practices with potential for harm.

## Data Availability

No datasets were generated or analysed during the current study.
